# Supplementary material for: Taxonomy of Micronesian monitors (Reptilia: Squamata: Varanus): endemic status of new species argues for caution in pursuing eradication plans
Source: R Soc Open Sci. 2020 May 13;7(5):200092. doi: 10.1098/rsos.200092 (PMC7277287; doi:10.1098/rsos.200092)
Supplement: Morphological data set [file rsos200092supp1.docx]

| **Species** | **Catalogue#** | **Locality** | **P** | **Q** | **XY** | **m** | **S** | **T** | **N** | **R** | **1** |
| --- | --- | --- | --- | --- | --- | --- | --- | --- | --- | --- | --- |
| *V. bennetti* | USNM212494 | Sarigan | 43 | 87 | 149 | 111 | 138 | 93 | 94 | 60 | 1.60 |
| *V. bennetti* | USNM122560 | Losiep Island | 41 | 79 | 156 | 105 | 140 | 98 | 90 | 60 | 1.89 |
| *V. bennetti* | USNM130186 | Yap Island | 41 | 94 | 154 | 98 | 131 | 97 | 92 | 63 | 1.79 |
| *V. bennetti* | USNM495369 | Ngcheangel atoll | 39 | 84 | 150 | 92 | 135 | 94 | 90 | 61 | 1.78 |
| *V. bennetti* | USNM495370 | Ngcheangel island | 39 | 74 | 151 | 97 | 129 | 88 | 87 | 55 | 1.76 |
| *V. bennetti* | USNM507504 | Ngeaur island | 45 | 90 | 158 | 109 | 138 | 100 | 92 | 64 | 1.82 |
| *V. bennetti* | USNM514125 | Ngeaur island | 45 | 87 | 155 | 105 | 137 | 93 | 94 | 60 | 1.70 |
| *V. bennetti* | USNM521719 | Ngeaur island | 41 | 87 | 160 | 97 | 135 | 98 | 91 | 63 | 1.85 |
| *V. bennetti* | AMNH 00624 | Yap Island | 46 | 87 | 152 | 85 | 137 | 97 | 95 | 68 | 1.60 |
| *V. bennetti* | AMNH 00625 | Yap Island | 45 | 92 | 153 | 96 | 143 | 97 | 92 | 70 | 1.67 |
| *V. bennetti* | AMNH 70652 | Koror | 44 | 86 | 161 | 103 | 138 | 93 | 90 | 62 | 1.80 |
| *V. bennetti* | AMNH 70653 | Koror | 42 | 83 | 150 | 98 | 130 | 95 | 90 | 65 | 1.79 |
| *V. bennetti* | ZMB17521 | Yap Island | 43 | 92 | 151 | 110 | 143 | 100 | 95 | 66 | 1.82 |
| *V. bennetti* | ZMB7819 | Yap Island | 49 | 96 | 152 | 96 | 132 | 96 | 91 | 61 | 1.78 |
| *V. bennetti* | ZFMK 45043 | ”Palau” | 44 | 87 | 160 | 107 | 139 | 100 | 88 | 55 | 1.68 |
| *V. bennetti* | ZMB17520 | Yap Island | 46 | 89 | 148 | 117 | 145 | 92 | 98 | 61 | 1.79 |
| *V. lirungensis* | MZB 5177 | Salibabu | 38 | 79 | 117 | 94 | 135 | 93 | 81 | 59 | 1.51 |
| *V. lirungensis* | MZB 5180 | Salibabu | 47 | 81 | 128 | 100 | 143 | 99 | 88 | 57 | 1.67 |
| *V. lirungensis* | MZB 5179 | Salibabu | 42 | 88 | 130 | 106 | 151 | 94 | 82 | 62 | 1.62 |
| *V. lirungensis* | MZB 581 | Karakelong | 39 | 87 | 126 | 101 | 147 | 98 | 87 | 60 | 1.75 |
| *V. lirungensis* | MZB 4195 | Karakelong | 41 | 85 | 126 | 95 | 150 | 95 | 81 | 57 | 1.60 |
| *V. lirungensis* | ZMA 15411a | Salibabu | 45 | 83 | 148 | 89 | 134 | 97 | 83 | 55 | 1.59 |
| *V. lirungensis* | ZMA 15411b | Salibabu | 43 | 82 | 159 | 87 | 145 | 101 | 84 | 60 | 1.45 |
| *V. lirungensis* | ZMA 15411a | Salibabu | 42 | 79 | 121 | 99 | 140 | 92 | 85 | 55 | 1.61 |
| *V. lirungensis* | ZMA 15411b | Salibabu | 41 | 81 | 122 | 103 | 139 | 101 | 84 | 65 | 1.43 |
| *V. rainerguentheri* | RMNH 3800 | Halmahera | 42 | 80 | 134 | 88 | 128 | 91 | 85 | 57 | 1.36 |
| *V. rainerguentheri* | RMNH 3190a | Halmahera | 43 | 78 | 136 | 88 | 120 | 93 | 92 | 57 | 1.40 |
| *V. rainerguentheri* | RMNH 3190b | Halmahera | 44 | 81 | 133 | 94 | 128 | 90 | 86 | 59 | 1.47 |
| *V. rainerguentheri* | ZMA 15417 | Halmahera | 43 | 76 | 140 | 85 | 127 | 94 | 87 | 58 | 1.52 |
| *V. rainerguentheri* | ZMA 15414a | Halmahera | 38 | 77 | 163 | 90 | 128 | 91 | 91 | 56 | 1.41 |
| *V. rainerguentheri* | ZMA 15414b | Halmahera | 43 | 82 | 155 | 89 | 139 | 93 | 85 | 61 | 1.45 |
| *V. rainerguentheri* | ZMA 15414c | Halmahera | 42 | 80 | 147 | 92 | 132 | 91 | 87 | 60 | 1.47 |
| *V. tsukamotoi* | USNM494383 | Cocos Island | 35 | 69 | 135 | 89 | 116 | 81 | 83 | 56 | 1.65 |
| *V. tsukamotoi* | USNM216368 | Guam | 33 | 74 | 135 | 92 | 118 | 85 | 82 | 53 | 1.57 |
| *V. tsukamotoi* | USNM494381 | Guam | 35 | 69 | 126 | 90 | 116 | 82 | 78 | 55 | 1.62 |
| *V. tsukamotoi* | USNM494382 | Guam | 33 | 68 | 130 | 86 | 118 | 78 | 76 | 53 | 1.67 |
| *V. tsukamotoi* | USNM507452 | Guam | 36 | 65 | 124 | 81 | 110 | 81 | 77 | 54 | 1.69 |
| *V. tsukamotoi* | USNM515827 | Guam | 35 | 67 | 130 | 85 | 116 | 85 | 81 | 57 | 1.67 |
| *V. tsukamotoi* | USNM515828 | Guam | 34 | 64 | 124 | 84 | 108 | 80 | 77 | 52 | 1.63 |
| *V. tsukamotoi* | USNM515829 | Guam | 33 | 65 | 133 | 85 | 113 | 82 | 77 | 57 | 1.63 |
| *V. tsukamotoi* | USNM521160 | Guam | 34 | 73 | 140 | 95 | 125 | 85 | 84 | 56 | 1.62 |
| *V. tsukamotoi* | USNM122462 | Guam | 32 | 66 | 129 | 80 | 114 | 83 | 80 | 53 | 1.65 |
| *V. tsukamotoi* | USNM216869 | Guam | 32 | 70 | 133 | 80 | 116 | 82 | 76 | 54 | 1.55 |
| *V. tsukamotoi* | USNM212491 | Pagan island | 34 | 66 | 136 | 80 | 113 | 83 | 79 | 57 | 1.67 |
| *V. tsukamotoi* | USNM212493 | Pagan island | 33 | 62 | 124 | 83 | 114 | 82 | 78 | 51 | 1.51 |
| *V. tsukamotoi* | AMNH 137202 | Rota Island | 37 | 68 | 137 | 85 | 116 | 87 | 84 | 56 | 1.64 |
| *V. tsukamotoi* | AMNH 137203 | Rota Island | 36 | 73 | 135 | 87 | 117 | 88 | 82 | 53 | 1.33 |
| *V. tsukamotoi* | AMNH 139987 | Rota Island | 36 | 71 | 132 | 86 | 106 | 85 | 77 | 58 | 1.48 |
| *V. tsukamotoi* | AMNH 139988 | Rota Island | 36 | 67 | 129 | 85 | 115 | 87 | 83 | 57 | 1.42 |
| *V. tsukamotoi* | USNM122652 | Rota Island | 36 | 71 | 133 | 83 | 113 | 85 | 80 | 57 | 1.57 |
| *V. tsukamotoi* | USNM285037 | Rota Island | 35 | 67 | 124 | 86 | 116 | 86 | 83 | 51 | 1.73 |
| *V. tsukamotoi* | USNM122653 | Rota Island | 33 | 61 | 126 | 80 | 101 | 86 | 75 | 52 | 1.47 |
| *V. tsukamotoi* | USNM576257 | Saipan island | 35 | 67 | 130 | 86 | 109 | 85 | 83 | 55 | 1.51 |
| *V. tsukamotoi* | USNM576258 | Saipan island | 35 | 63 | 129 | 86 | 111 | 84 | 81 | 51 | 1.65 |
| *V. tsukamotoi* | USNM576259 | Saipan island | 33 | 65 | 131 | 88 | 108 | 85 | 81 | 55 | 1.54 |
| *V. tsukamotoi* | USNM212487 | Saipan island | 32 | 66 | 133 | 88 | 113 | 83 | 79 | 55 | 1.61 |
| *V. tsukamotoi* | USNM308067 | Tinian island | 36 | 64 | 135 | 92 | 112 | 83 | 78 | 53 | 1.69 |
| *V. tsukamotoi* | USNM124112 | Yaptan Island | 38 | 73 | 133 | 95 | 113 | 84 | 78 | 57 | 1.42 |
| *V. tsukamotoi* | USNM124113 | Yaptan Island | 35 | 74 | 134 | 93 | 112 | 88 | 81 | 52 | 1.68 |
| *V. tsukamotoi* | AMNH 78994 | Yaptan Island | 35 | 64 | 130 | 76 | 118 | 88 | 75 | 48 | 1.42 |
| *V. tsukamotoi* | MNHN 1888 20 | Saipan island | 35 | 58 | 117 | 85 | 104 | 83 | 84 | 48 | 1.54 |
